# Event and Comment

**Published:** 1916-12

**Authors:** 


### DENTAL REGISTER

Vol. LXX DECEMBER, 1916 No. 12

### EVENT AND COMMENT

SURPRISING DENTAL FINANCING. In the *Western Dental Journal* for November is printed the treasurer's report of the Tri State Dental Study Club meeting held in Kansas City last March. It cites the main facts that a total of $6,649.71 was received from dentists attending, exhibits, and loans of $100 each from the three state societies interested. The expenses of promotion and conducting the meeting were $3,368.84; the cost of instruction was $1,950; $300 borrowed money was returned; and $1,868.87 was divided between the three state societies as profits. Now we call this pretty good management so far as money making in dental education is concerned. The profits of running a dental college can never again be held up in dental society debates, as a grasping monopoly out only for the money there is in the business of education. We have no doubt every man who attended got more for his money than he expected and is satisfied with his investment, for they had our brightest men there as teachers. We are a bit curious though how these teachers feel about the amount they received for their work, which by the way was all there really was that was of real value. Some of these teachers traveled long distances, and their business at home ceased to produce income, and expenses went on just the same. With the $264 each teacher received, he certainly was not over paid for his loss of time and opportunity to earn, to say nothing of the years of time and effort he had to spend to qualify himself to fill so important a position. We can't help feeling that the $1,800 turned back to the state treasuries of the three state socities, properly belongs to these teachers, who surely earned it.

Another matter which seems to us surprising, is that it should have cost over $3,000 to promote and conduct such a meeting. But the items are all there: such as printing bills over $637, theatre $267, banquet $250, expenses of committeemen $300, badges $171 (some badges!), and other things to make up the grand total. They surely do things in the large out West.

DENTISTRY IN THE EUROPEAN WAR. It may not be fully realized by us what demands the great European war is making on the dental profession in those countries where the activities are greatest. The terrible trench fighting is productive of the most horrible mutilation of the heads and faces of the soldiers, and jaw and face restorations of the most intricate character are in great demand. The American Ambulance Hospital of Paris, which is wholly sustained by Americans, has attached to it as one of its most important services, a Dental Department. The Dental Department is under the control of two American dentists, Dr. George B. Hays and Dr. Davenport, both for many years well known practitioners of dentistry in Paris. These men have sacrificed their practices and are giving practically all their time to this relief work without monetary compensation of any sort. They have had some support in contributions of money and materials, and help from American practitioners who have gone there for three months service at their own charges. But it is getting difficult to keep enough experienced dentists coming to take care of the increasing demands which the war is causing, and which the effective service rendered by the dental department is developing. We are printing in this issue a private letter acknowledging the receipt of a box of instruments and materials sent by Dr. L. P. Hall, of Ann Arbor, last summer. The letter is from Dr. Hays, and makes a personal appeal for help in this work to his American conferers that is almost irresistible.

The character of the service and the professional benefits to be had ought to appeal so strongly to specialists in oral surgery and oral prosthesis, that it would seem sufficiently attractive to enlist the co-operation of enough such men to supply all the help of this kind needed. Such an experience would certainly be of incalculable value to such practitioners, to say nothing of the humanitarian appeal from the great number of sufferers. It is not inexperienced graduates that are needed, but men of skill and experience, who can take advantage of the opportunities and successfully cope with the unusual conditions resulting from the terrible warfare methods.

It seems too bad that the United States War Department can not see the advantage of sending to Europe the best dental surgeons in our army to study the methods of caring for jaw and head wounds; as we may some day have just this kind of service to render in our own country. We are writing this comment and printing the extract from the personal letter above referred to in the hope that it may attract someone to this most worthy work, or that it may interest some who can not go, or serve, to send some financial contribution that is also much needed. We shall be very glad to correspond with anyone who may be interested in going, or in sending help of any kind, or of any amount of money. Any kind of money will serve, but only the skillful can meet the emergency in person.

EXTRACT FROM DR. HAY'S LETTER.

To D. L. P. Hall, Ann Arbor, Mich.

*My Dear Doctor Hall*—Your second generous gift has arrived, and I am glad to tell you that it has given us all the joys of a Christmas box (this was a box of dental supplies). I assure you we are gratefully appreciative of your thoughtfulness, and I extend to you the hearty thanks of the entire department for your most appropriate selection of useful instruments and materials. Everything you sent can and will be put to good use immediately, and your things will be a great saving to us in the running expenses of the department.

I am so pressed with the work here, and the little time that I am forced to give to my private practice, that my days are full from early morning until after seven o'clock each day, and my evenings practically do not exist, as I find the only way I can keep going is to retire as soon as possible after dinner for a long night of rest and recuperation for another strenuous day. How long this may continue, I can't of course say, but I am giving up everything to be able to keep this work going, and I trust I shall have the physical strength to do this.

We are keeping the work going but with great sacrifice and much annoyance, because of the short time our volunteers are with us. Thus far the American helpers have remained with us for three months only. It is difficult and troublesome to be getting new men over so frequently, and equally troublesome to break in new men, especially those who lack experience along the lines of work needed. Three of our men are leaving next month and we do not now know who we can secure to replace them, not being in touch with the men in the states. Because of my long absence in this country I do not know to whom I could appeal. Dr. Guilford, of Philadelphia, has helped us heretofore, but now he has no one to send us. I know all the good men at home have their own affairs to look after, but I am hoping you may know of someone who could be prevailed upon to come over, because of the wonderful advantages he could have here for personal development, and which probably never will be offered again. I am sure there are many men who would come if they knew what was to be seen and had here. Can't you send us someone from the university?

I have declined the services of a good many young men, they are of no great value to us because of their lack of experience. We have had such remarkable success and have earned and received such satisfactory commendation for our work that we do not want to hazard our reputation with unskillful operators. The work is intricate and exacting, and will test out the skill of the very best men, and it is to this class that we appeal for help. We are having the services of the best American surgeons continually, and they have expressed their interest in our dental work and have co-operated with us most freely and cordially. Naturally we want to hold up our end as nearly on an equality with the medical men as possible. To do this you must send us your best men, and also because the work requires full grown men. So don't hesitate to commend it to the very best men you have. I am confident anyone who comes will not regret the experience. If you can devise some way of helping us out in this matter you will do a great favor to humanity and the cause for which we are working.

With warmest personal regards, and most cordial and sincere appreciation of all your favors, and again thanking you for your appropriate remembrance, I am,

Most sincerely yours,
GEORGE B. HAYES, D. D. S.

Paris, October 27, 1916.

